# H_2_S-induced pancreatic acinar cell apoptosis is mediated *via* JNK and p38 MAP kinase

**DOI:** 10.1111/j.1582-4934.2008.00318.x

**Published:** 2008-03-29

**Authors:** Sharmila Adhikari, Madhav Bhatia

**Affiliations:** Department of Pharmacology, National University of SingaporeSingapore

**Keywords:** H2S, apoptosis, ERK, JNK, p38

## Abstract

Treatment of pancreatic acinar cells by hydrogen sulphide has been shown to induce apoptosis. However, a potential role of mitogen-activated protein kinases (MAPKs) in this apoptotic pathway remains unknown. The present study examined the role of MAPKs in H_2_S-induced apoptosis in mouse pancreatic acinar cells. Pancreatic acinar cells were treated with 10 μM NaHS (a donor of H_2_S) for 3 hrs. For the evaluation of the role of MAPKs, PD98059, SP600125 and SB203580 were used as MAPKs inhibitors for ERK1/2, JNK1/2 and p38 MAPK, respectively. We observed activation of ERK1/2, JNK1/2 and p38 when pancreatic acini were exposed to H_2_S. Moreover, H_2_S-induced ERK1/2, JNK1/2 and p38 activation were blocked by pre-treatment with their corresponding inhibitor in a dose-dependent manner. H_2_S-induced apoptosis led to an increase in caspase 3 activity and this activity was attenuated when caspase 3 inhibitor were used. Also, the cleavage of caspase 3 correlated with that of poly-(ADP-ribose)-polymerase (PARP) cleavage. H_2_S treatment induced the release of cytochrome *c*, smac from mitochondria into the cytoplasm, translocation of Bax into mitochondria and decreased the protein level of Bcl-2. Inhibition of ERK1/2 using PD98059 caused further enhancement of apoptosis as evidenced by annexin V staining, while SP600125 and SB203580 abrogated H_2_S-induced apoptosis. Taken together, the data suggest that activation of ERKs promotes cell survival, whereas activation of JNKs and p38 MAP kinase leads to H_2_S-induced apoptosis.

## Introduction

Mitogen-activated protein kinases (MAPK) superfamily consist of three family members: the extracellular signal-regulated kinases (ERK), c-jun N-terminal kinase (JNK) and p38 [1]. ERKs, JNKs and p38 MAP kinase are structurally related, and all of them are activated by phosphorylation of threonine and tyrosine. These play crucial roles in determining whether the cell dies or survives (proliferation or apoptosis) [2]. ERK 1 and 2 are part of the ras/raf/MEK pathway often associated with proliferation and survival [3].ERK-regulating kinase (MEK) has been shown to activate ERKs, but MEK independent activation has also been reported [4, 5]. The other MAP kinases (JNK and p38) have been implicated primarily in the induction of apoptosis and inflammation after exposure to different agents [6, 7]. However, several isoforms of JNK and p38 have been identified that may have different functions and localization. Also, some reports have indicated that more complex roles of these pathways may exist to transmit other ultimately distinct cellular effect in different cell lineages [8, 9]. Although MAPKs have been reported to be activated in different cell lines, including neonatal cardiomyocytes [10], NIH 3T3 cells [11], KB-3 carcinoma cells [12], H9 leukaemia cells [13], human lung adenocarcinoma H522 cells [14] and neuroblastoma cells [15], the role of MAPKs varied depending on the different cell types. Treatment of pancreatic acinar cells by H_2_S induces acinar cell apoptosis [16] and it has also been reported that H_2_S-induced hypoxia triggers the proliferative phases of cell cycle entry in non-transformed rat intestinal epithelial cells [17]. However, it was noted that a higher concentration of H_2_S induced late apoptotic events in these cells [17]. Also pro-inflammatory effect of H_2_S by substance P-neurokinin 1 receptor pathway has been reported in pancreatic acinar cells [18]. Apoptosis is executed by a subfamily of cysteine proteases known as caspases. In mammalian cells, a variety of apoptotic stimuli induce a series of biochemical reactions that result in caspase activation [19]. Caspase 3 is an executioner of apoptosis by cleavage of several essential cellular proteins such as poly-(ADP-ribose)-polymerase (PARP) [20]. Mitochondria play a central role in cellular metabolism and apoptosis. During apoptosis, a wide variety of cellular signals elicited from the membrane, cytosol or mitochondria are activated by stimuli. These signals can disturb energy metabolism and modulate the expression of Bcl-2 family members [21]. Mitochondria may sense the death signals and commit cells to apoptosis by releasing death factors into the cytosol, such as cytochrome *c*. cytochrome *c* plays roles in triggering apoptosis and in the activation of downstream caspases to cleave cellular substrates [22, 23]. Bax is a pro-apoptotic member of the Bcl-2 family. Accumulated evidence shows that death signals induce a conformational change of Bax, leading to its mitochondrial translocation [24, 25]. On the other hand, Bcl-2 family proteins such as Bcl-2 prevent the occurrence of apoptosis via regulating mitochondrial homeostasis and blocking cytochrome *c* release and caspase activation [26].

In the present study, we examined the activation of ERK1/2, JNK1/2 and p38 kinases in isolated pancreatic acinar cells exposed to H_2_S. In order to describe their involvement in H_2_S-induced apoptosis, we also examined the annexin V binding, followed by caspase 3 activities as well as the levels of pro- and anti-apoptotic proteins. We found that activation of ERK is required for promoting cell survival whereas activation of JNKs and p38 MAP kinase is critical for induction of apoptosis.

## Materials and methods

### Animals

All experimental procedures were performed in accordance with the Guide for the Care and Use of Laboratory Animal (NIH Publication, 1996) and approved by the animal ethics committee of National University of Singapore. Swiss mice (male, 25–30 g) were housed in a controlled environment with an ambient temperature of 22–26°C and a 12 hrs light/dark cycle. They were fed with standard laboratory chow and given water ad libitum.

### Preparation of pancreatic acini

Pancreatic acini were obtained from mouse pancreas by collagenase treatment. Briefly, pancreata from mice were infused with buffer A (140 mM NaCl, 4.7 mM KCl, 1.13 mM MgCl_2_, 1 mM CaCl_2_, 10 mM glucose, 10 mM Hepes, pH 7.2) containing 200 IU/ml collagenase, and incubated in a shaking water bath for 10 min. at 37°C. The digested tissue was passed through 50 mg/ml bovine serum albumin (BSA), and washed twice with buffer A for further experiments. Cell viability was determined by trypan blue exclusion.

### Induction of pancreatic acinar cell apoptosis and treatment with inhibitors

The prepared acini were distributed into microfuge tubes containing buffer A. NaHS (a donor of H_2_S) was added into these tubes with the working concentration of 10 μM. Acini were incubated with or without NaHS at 37°C in a shaker water bath for 3 hrs. In some experiments, selective MEK1 inhibitor PD98059 at 10 μM, 30 μM or 50 μM, JNK inhibitor SP600125 at 1 μM, 5 μM or 10 μM and p38 inhibitor SB203580 at 10 μM, 30 μM or 50 μM (calbio-chem-Behring, Za Jolla, CA, USA) were added into fresh buffer of pancreatic acinar cells for 30 min. before NaHS treatment. The cell pellet was used for Western blot analysis. PD98059, SP600125 and SB203580 stock solutions were prepared by dissolving 5 mg of PD98059, SP600125 and SB203580 into 100 μl of dimethyl sulfoxide (DMSO). The final concentration of the vehicle was 0.1% DMSO.

### Preparation of total cell lysates for Western blot analysis

After treatment, pancreatic acinar cells were homogenized on ice in RIPA buffer supplemented with 1 mM phenylmethylsulfonyl fluoride (PMSF) and the protease inhibitor cocktail containing pepstatin, leupeptin, chymostatin, antipain and aprotinin (5 μg/ml of each), and centrifuged at 4°C for 15 min. at 14,000 ×*g*. The supernatants were collected and stored at −80°C until use. Protein concentrations were determined by the Bio-Rad protein assay (Bio-Rad Laboratories, Hercules, CA, USA).

### Western blot analysis

Cell lysates (50 μg) were fractionated using 4–20% SDS-polyacrylamide gel and electrophoretically transferred and blotted onto polyvinylidene fluoride (PVDF) membranes using the manufacturer's standard protocol (Invitrogen Corporation, Carlsbad, CA, USA). Non-specific binding was blocked by 1 hr incubation of the membranes in 5% non-fat dry milk in phosphate buffered saline Tween-zo (PBST) (0.05% Tween 20 in PBS). The blots were then incubated overnight with the primary antibodies phospho-ERK1/2, ERK 1/2, phospho-SAPK/JNK, SAPK/JNK, phospho-p38, p38, Caspase 3 (Cell Signaling Technology, Danvers, MA, USA) PARP (Roche Molecular Biochemicals, Mannheim, Germany), hypoxanthine-guanine phosphoribosyl transferase (HPRT), Bcl-2, smac, cytochrome *c* (Santa Cruz Biotechnology Santa Cruz, CA, USA) and Bax (Chemicon International, Inc., Tenecula, CA, USA. The above antibodies were used at 1:1000 dilutions in the buffer containing 2.5% non-fat dry milk in PBST. After which they were washed four times with PBST, and finally incubated for 1 hr with goat anti-rabbit horseradish peroxidase (HRP)-conjugated secondary antibody (Santa Cruz Biotechnology) at 1:2000 dilutions in the buffer containing 2.5% non-fat dry milk in PBST. The blots were developed for visualization using enhanced chemiluminescence (ECL) detection kit (Pierce, Rockford, IL, USA). The densities of the band were quantified using Lab works image analysis software.

### Detection of caspase 3 activity in live cells

Pancreatic acinar cells were treated with NaHS at 10 μM concentration for 3 hrs at 37°C. In some experiments, caspase 3 inhibitor Ac-DEVD-CHO was used together with crambene. After treatment, the cells were analysed for caspase 3 activity using NucView™ 488 Caspase-3 assay kit for live cells (Biotium, CA, USA). Briefly the treated cells were incubated with 0.2 mM NucView™ 488 Caspase 3 substrate for 15 min. Cell staining was observed under a fluorescence microscope using fluorescein isothio-cyanate (FITC) filter.

### Isolation of cytosol and mitochondria

Mitochondria were isolated from NaHS-treated pancreatic acinar cells using the mitochondria isolation procedure of the Pierce protocol (Pierce Biotechnology, Rockford, IL, USA). The pellet with mitochondria was lysed as described earlier for the whole cell lysates. The cytosolic and mitochondrial fractions were analysed by Western blot using smac, cytochrome *c* and Bax antibodies as described above.

### Annexin V-FITC staining detection

Extent of apoptosis was determined by Annexin V-FITC staining using BD ApoAlert™ Annexin V-FITC apoptosis Kit. Treatment of acinar cells was carried out as described above. After treatment, the acini were incubated with 5 μl Annexin V (20 μg/ml in Tris-NaCl) and in 1× binding buffer for 15 min. in the dark at room temperature. Samples were quantified by Gemini EM Microplate Spectrofluorometer measuring green fluorescence (excitation 488 nm, emission 530 nm). Data were expressed as relative fluorescent unit per μg DNA per μl.

### Statistical analysis

All experiments were repeated at least three times. Results are expressed as mean **±** SE. The significance of changes was evaluated by using anova and Tukey's method was used as a post hoc test for the difference between groups. A *P* value ≤ 0.05 was taken as the level of significance.

## Results

### Activation of MAPKs by NaHS

**Fig. 1:**
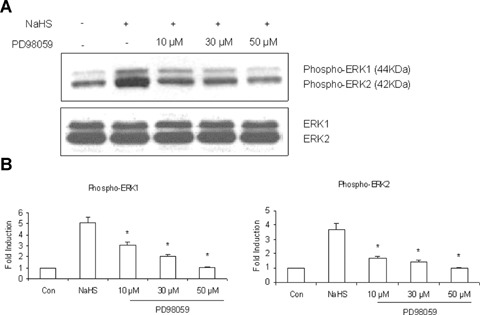
The expression of phosphorylated and total ERK in pancreatic acinar cells. (**A**) Western blot analysis of NaHS-induced pancreatic acinar cells pre-treated for 30 min. with or without PD98059 at the indicated concentrations (10 μM, 30 μM or 50 μM) then incubated with 10 μM NaHS for an additional 3 hrs. Cell lysates were resolved in 4–20% SDS-polyacry-lamide gels and the blots were incubated with specific antibodies against the active phosphorylated forms of ERK. Loading equality was confirmed with antibodies of total ERK. Representative data of three independent experiments are shown. (**B**) Densitometric analysis of phospho-ERK1/2 and total ERK1/2. Data presented are means **±** SD. *P≤ 0.05 statistically significant comparison with that of NaHS-treated group.

To examine whether MAPKs are activated upon NaHS treatment in pancreatic acinar cells, the phosphorylation of MAPKs were analysed. Mouse pancreatic acinar cells were treated with 10 μM NaHS for 3 hrs at 37°C. Cells were then lysed and cell proteins were subjected to Western blot analysis using antibodies against both phospho-ERK1/2 and total ERK1/2. To verify whether the MEK1 inhibitor PD98059 blocks phosphorylation of ERK1/2 in NaHS-treated cells, mouse pancreatic acinar cells were pre-incubated with PD98059 for 30 min. followed by stimulation with 10 μM NaHS for 3 hrs. As shown in Fig. 1A, NaHS-induced phosphorylation of ERK1/2 in pancreatic acini and PD98059 attenuated NaHS-induced phosphorylation of ERK1/2 in a dose-dependent manner. Densitometric analysis of Western blot experiments revealed a significant increase in phosphorylation of ERK1/2 and three different doses of PD98059 10 μM, 30 μM and 50 μM significantly inhibited the phosphorylation of ERK1/2 when compared to NaHS only treated group (Fig. 1B).

**Fig. 2:**
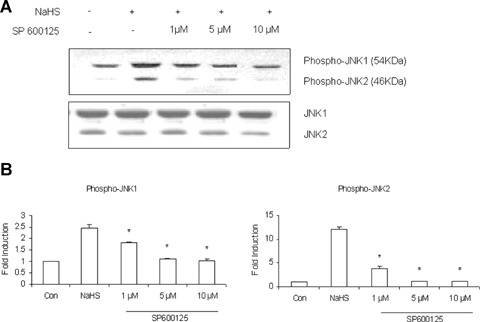
The expression of phosphorylated and total JNK in pancreatic acinar cells. (**A**) Pancreatic acinar cells were treated with 10 μM NaHS for 3 hrs and pre-incubated for 30 min. with or without 1 μM, 5 μM or 10 μM of the selective JNK1/2 inhibitor SP600125. Western blots showing the phospho-JNK1/2 and total JNK1/2. NaHS-induced phosphorylation of JNK was prevented by selective inhibition of SP600125. Representative data of three independent experiments are shown. (**B**) Intensities of phospho-JNK1/2 and total JNK1/2 were determined by den-sitometry. Values are mean **±** SD. *P≤ 0.05 versus NaHS-treated group.

To examine whether NaHS stimulates JNK phosphorylation in pancreatic acini, whole cell lysates were prepared and analysed using antibodies against both phospho-JNK1/2 and total JNK1/2. To confirm that SP600125, an inhibitor of JNK which prevents the phosphorylation of JNK substrates by blocking the adenosine-5′-triphosphate (ATP)-binding domain of JNKs, inhibits phosphorylation of JNK in NaHS-treated cells, mouse pancreatic acinar cells were pre-incubated with SP600125 (1 μM, 5 μM or 10 μM) for 30 min. followed by stimulation with 10 μM NaHS for 3 hrs. As shown in Fig. 2A, NaHS-induced phosphorylation of phospho-p54 and phospho-p46 JNK in pancreatic acini and SP600125 attenuated NaHS-induced phosphorylation of JNK. In Fig. 2B, densitometric analysis of Western blot experiments revealed a significant increase in phospho-p54 and phospho-p46 when compared to control and SP600125 significantly blocked phosphorylation of JNK1/2 when compared to NaHS treated group in a dose-dependent manner.

**Fig. 3:**
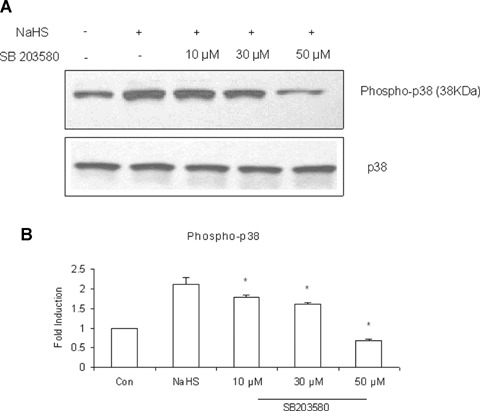
The expression of phosphorylated and total p38 in pancreatic acinar cells. (**A**) Pancreatic acinar cells were treated with or without SB203580 (10 μM, 30 μM or 50 μM) for 30 min. followed by treatment with 10 μM NaHS for 3 hrs. Cell lysates were prepared. Fifty microgram of total protein was used for Western analysis using an antibody recognizing phosphorylated and total p38. Total p38 was used to normalize protein loading. Results are characteristic of three independent experiments. (**B**) Quantitation of the data shown in panel A. Values represent means **±** SD. *P ≤ 0.05 statistically significant comparison with that of NaHS-treated group.

In order to determine the involvement of p38 phosphorylation in NaHS-treated pancreatic acini, whole cell lysates were prepared and analysed using antibodies against both phospho-p38 and total-p38. To verify whether the p38 inhibitor SB203580 blocks phosphorylation of p38 in NaHS-treated cells, mouse pancreatic acinar cells were pre-incubated with SB203580 for 30 min. followed by stimulation with 10 μM NaHS for 3 hrs. As shown in Fig. 3A, NaHS-induced phosphorylation of p38 in pancreatic acini and SB203580 attenuated NaHS-induced phosphorylation of p38 in a dose-dependent manner. Densitometric analysis of Western blot experiments revealed a significant increase in phosphorylation of p38 and three different doses of SB203580, 10 μM, 30 μM and 50 μM significantly blocked phosphorylation of p38 when compared to NaHS only treated group (Fig. 3B).

## Caspase 3 activity and PARP cleavage

**Fig. 4:**
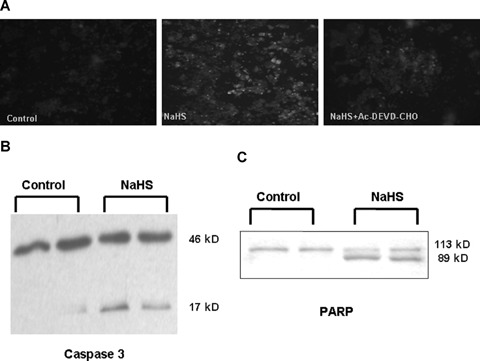
NaHS-induced apoptosis is caspase dependent. (**A**) Caspase 3 activity detection by fluorescence microscopy. NucView™ 488 Caspase -3 substrate consists of a fluorogenic DNA dye and a DEVD substrate moiety specific for caspase 3. The released DNA dye migrates to the cell nucleus to stain the nucleus bright green. Treatment with caspase 3 inhibitor Ac-DEVD-CHO inhibits caspase 3 activity. Photographs visualized with magnification ×400. (**B**) Representative Western blot indicates cleavage of caspase 3 migrating at approximately 17 kD, following treatment with 10 μM NaHS. (**C**) Western blot indicates cleavage of poly-(ADP-ribose)-polymerase (PARP) which correlates with the activation of caspase 3. Note that the antibody recognizes both uncleaved PARP (113 kD) and the larger cleavage fragment (89 kD).

Caspase 3 activity was evaluated in NaHS-induced pancreatic acinar cells. Pancreatic acinar cells were treated with or without NaHS at the concentration of 10 μM for 3 hrs and the caspase activity was determined. As shown in Fig. 4A, NaHS triggered caspase 3 activation as evidenced by fluorescence microscopy when compared to that of untreated cells. Also, treatment with the caspase 3 inhibitor Ac-DEVD-CHO attenuated caspase 3 activity. The activation of caspase 3 was also confirmed by Western blot analysis. NaHS induced the cleavage of caspase 3 in pancreatic acinar cells (Fig. 4B). In a parallel experiment, treatment of pancreatic acinar cells with NaHS induced the PARP cleavage. NaHS-induced cleavage of caspase 3 correlated with the cleavage of its substrate PARP (Fig. 4C). These results suggest that H_2_S-induced apoptosis is caspase dependent.

### Changes in Bax protein level, induction of smac and cytochrome *c* release and Bcl-2 expression

**Fig. 5:**
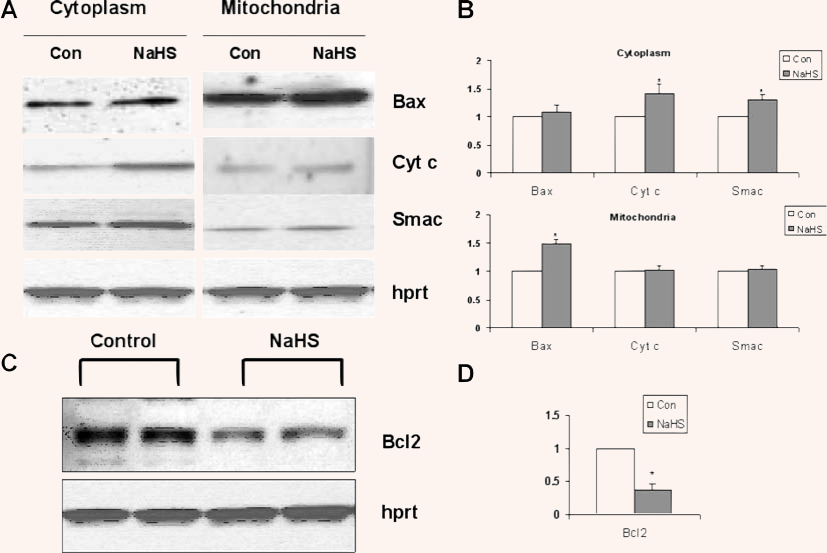
NaHS induced the translocation of Bax, release of cytochrome c and smac and decrease in Bcl-2 expression. (**A**) Aliquots containing equal amounts of protein from cytosolic and mitochondrial fractions were subjected to Western blot analysis. NaHS-induced translocation of Bax from the cytosolic to mitochondrial fractions and release of cytochrome c and Smac into the cytoplasm. Hypoxanthine-guanine phosphoribosyl transferase (HPRT) was used as a loading control. (**B**) Intensities of Bax, cytochrome c and smac were determined by densitometry. Values are mean **±** SD of three independent experiments. *P≤ 0.05 *versus* control group. (**C**) Changes in the expression of anti-apoptotic protein Bcl-2. NaHS treatment reduces the expression of Bcl-2 protein levels. (**D**) Protein bands were quantified by densitometry and the changes are represented in the graph. Values represent means **±** SD of three independent experiments. *P≤ 0.05 *versus* control group.

To determine whether the NaHS-induced cell death was associated with changes in the function of the Bcl-2 family, Western blot analysis of apoptosis signalling proteins were carried out. In this study, we isolated mitochondria and measured the translocation of smac, cytochrome *c* and Bax by Western blot in both mitochondria and cytosolic fractions. Cells were treated with NaHS (10 μM) for 3 hrs. As shown in Fig. 5A and B, smac and cytochrome *c* was released into the cytoplasm. In addition, NaHS treatment of pancreatic acinar cells led to an increased translocation of Bax from the cytosol to the mitochondria, indicating that NaHS may act via the Bax and smac and cytochrome *c* pathway to induce apoptotic cell death. On the other hand, we also investigated whether NaHS treatment can modulate the expression of anti-apoptotic protein Bcl-2. Western blot analysis revealed that NaHS significantly down-regulated Bcl-2 in whole cell lysates (Fig. 5C and D).

### Effects of ERK, JNK and p38 on NaHS-induced apoptosis

**Fig. 6:**
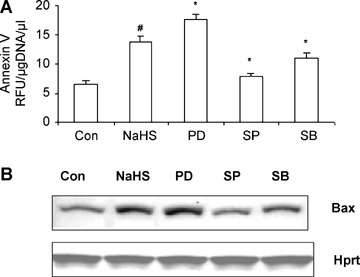
The relationship of MAPK activation in NaHS-induced pancreatic acinar cell apoptosis. Pancreatic acinar cells were pre-treated with or without PD98059 (30 μM), SP 600125 (5 μM) or SB203580 (30 μM) for 30 min. prior to the addition of 10 μM NaHS. (**A**) Samples were quantified using a fluorescent plate reader with a FITC filter (excitation 488 nm; emission 530 nm) for annexin V binding. Data were expressed as relative fluorescent units (RFU) per microgram DNA per microlitre. *#P*≤ 0.05 when compared to control, *P≤ 0.05 when compared to NaHS-treated group. (**B**) Effect of inhibitors on the changes of bax expression was analysed by Western blot analysis. HPRT was used as a loading control. The results represent three independent experiments.

To analyse the consequence of the activation of ERK, JNK and p38 MAP kinase induced by NaHS, we measured annexin V binding, a marker for early stage apoptosis. Fluorescence measurement using a plate reader showed that annexin V FITC binding was statistically elevated in NaHS-treated cells compared with untreated control (Fig. 6A). Pancreatic acinar cells were pre-treated with or without PD98059 (30 μM), SP600125 (5 μM) or SB203580 (30 μM) for 30 min. followed by treatment with 10 μM NaHS for 3 hrs. The concentration selected for each inhibitor showed a significant inhibition of MAPKs. PD98059 showed a significant increase in annexin V FITC binding when compared to NaHS-treated group. On the other hand, SP600125 which inhibits JNK and SB203580 which inhibits p38 led to a significant decrease of annexin V FITC binding when compared to NaHS-treated group. Also, PD98059 showed an increase in bax expression when compared to NaHS-treated group. On the other hand, SP600125 and SB203580 led to a decrease in bax expression when compared to NaHS-treated group (Fig. 6B). These results indicate the involvement of JNK and p38 MAPK cascades in NaHS-induced apoptosis.

## Discussion

Recent evidence indicates that the MAPK family protein kinases are important mediators of apoptosis induced by stressful stimuli [27, 28]. The present study demonstrates that treatment of pancreatic acinar cells by H_2_S are capable of activating the phosphorylation of MAP kinase signalling pathways with the result of an activation of apoptotic signalling cascade and apoptosis. To gain insight into the signalling mechanisms involved in pancreatic acinar cell apoptosis treated by H_2_S, we examined the activation of ERK1/2, JNK1/2 and p38 kinases. All the three MAPKs, ERK1/2, JNK1/2 and p38 were activated after H2S treatment. Moreover, this activation was blocked in a dose-dependent manner when their corresponding inhibitors were used. Inhibition of ERK with PD98059 caused further enhancement of apoptosis, whereas inhibition of JNK with SP600125 or p38 MAPK with SB203580 were associated with a decrease in apoptosis.

It has been reported that H_2_S induces apoptosis of insulinsecreting **β** cells by enhancing endoplasmic reticulum stress via p38 MAPK [29]. In human aorta smooth muscle cells, ERK played an active role in mediating apoptosis [30]. Among MAPK subfamilies, ERK is controversial in its role in cell death. While some studies showed that the ERK activation mediates a survival response that counteracts cell death, other studies reported that the ERK activation is associated with apoptotic signalling pathways [31]. The data presented here suggests that activation of ERKs may promote cell growth, whereas activation of JNKs and p38 MAP kinase is associated with acinar cell apoptosis. This indicates that ERKs counteract JNKs and p38 MAP kinase. Cross-talk between different signal transduction pathways is a basic regulatory mechanism in the cells. The pathways leading to the activation of ERKs and JNKs consist of multiple signalling components, most of which are regulated by protein phosphorylation and there are multiple ways for the cross-talk to take place. Activation of JNKs and p38 MAP kinase appears to counteract the activation of ERKs, and the dynamic balance between growth factor-activated ERKs and stress-activated JNK-p38 MAP kinase pathways is thought to be important in determining cell survival or apoptosis [32].

Apoptosis serves as a major mechanism for the precise regulation of cell numbers and as a defence mechanism to remove unwanted and potentially dangerous cells. The apoptosis pathway involves multiple components, and a central element of the pathway is the Bcl-2 family of proteins [33–35]. The balance between pro-apoptotic and anti-apoptotic members of Bcl-2 family of proteins determines the ability of cells to either survive or undergo apoptosis after certain stimulus or injury. This study indicates that NaHS-induced apoptosis in pancreatic acinar cells is mediated by Bcl-2 down-regulation, which subsequently activates caspase 3, an important effector caspase followed by cleavage of PARP for apoptosis. Bcl-2 and inhibitor of apoptosis (IAP) proteins have been investigated as potential therapeutic targets on the basis of their ability to disrupt apoptosis and to confer resistance to chemotherapy in cancer cells [36, 37]. Bcl-2 can form ion channels in biological membranes [38], and those channels may control apoptosis by influencing intracellular membranes permeability and smac and cytochrome *c* release from mitochondria [39]. Activation of p38 kinase by adriamycin was also found to play a pro-apoptotic role [10]. In this regard, adriamycin has been shown to cause cytochrome *c* release from the mitochondria and cytochrome *c* release is known to precede apoptosis [40]. Mitochondria play essential roles in apoptosis through the re-distribution of intermembranous mitochondrial proteins such as cytochrome *c*[41]. Bax translocation plays a critical role in the mitochondria-induced release of cytochrome *c*[24]. However, treatment of human aorta smooth muscle cells with H_2_S did not significantly alter the expressions of Bax and Bcl-2 [30]. The physiological role of PARP cleavage during apoptosis may serve as an energy conserving step enabling the cell to complete the process of apoptosis [42]. Also, PARP cleavage prevents induction of necrosis and ensures appropriate execution of caspase-mediated programmed cell death [43].

**Fig. 7:**
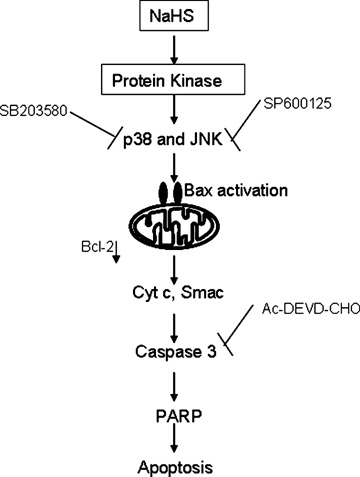
Schematic diagram of the signalling pathways of NaHS-induced pancreatic acinar cell apoptosis. The signalling pathways proposed by our experimental data are summarized. NaHS induces the activation of p38 and JNK. NaHS decreases the level of anti-apoptotic protein Bcl-2, induces the translocation of Bax, release of cytochrome c and smac and activation of caspase 3 and its substrate PARP leading to cell death.

Based on the data presented in this study, a generalized sequence of events is proposed (Fig. 7). H_2_S causes activation of JNK1/2 and p38 kinases and this activation is inhibited when SP600125 and SB203580 were used and also it inhibits H_2_S-induced apoptosis. On the other hand, ERK inhibitor PD98059 enhances apoptosis. These data also correlates with the alterations of Bax expression. PD98059 shows an increase in Bax protein levels whereas SP600125 and SB203580 show a decrease in Bax expression. High expression of Bax might cause activation of cell death signalling cascade. Each of these MAPKs has unique role in the regulation of cellular metabolism and gene expression related to growth, apoptosis and cellular responses to external stresses [44]. Our data for the first time suggests that H_2_S-induced apoptosis is mediated via JNK and p38 MAPK. In conclusion, our findings may provide insight into the mechanism involved in the apoptotic pathway and to support the idea that H_2_S could induce apoptosis in mouse pancreatic acinar cells through the JNK and p38 MAPK. However, further studies are necessary in order to investigate what kind of molecular pathways are related to H_2_S-induced apoptosis.

Evidence is accumulating to demonstrate that inhibitors of H_2_S production or therapeutic H_2_S donor compounds exert significant effects in various animal models of inflammation, reperfusion injury and circulatory shock [45]. Cai *et al*. have shown that Akt phosphorylation was significantly increased upon H_2_S treatment [46]. On the other hand, excessive production of H_2_S may contribute to the pathogenesis of inflammatory diseases, septic shock, cerebral stroke and mental retardation in patients with Down syndrome and reduction of its production may be potential therapeutic value in these states [47]. In the cardiovascular system, H_2_S relaxes vascular smooth muscles by the activation of KATP channels and inhibits smooth muscle cell proliferation via the mitogen-activated protein kinase signalling pathway [48]. Additional studies are needed to delineate the various interplays between the gasotransmitters in health and disease, and to identify areas in which pharmacological modulation of these agents (alone or in combination) may provide therapeutic benefit.
